# Serum profile of IL-29, IL-41, and raftlin in microvascular complications of type 2 diabetes mellitus

**DOI:** 10.1038/s41598-026-55654-y

**Published:** 2026-06-16

**Authors:** Noorulhuda F. Khalaf, Isam N. Salman, Ali H. Ad’hiah, Nor Effa S. Zulkafli

**Affiliations:** 1 Department of Biomedical Sciences, Pusat Kanser Tun Abdullah Ahmad Badawi, Universiti Sains Malaysia, 13200 Bertam, Penang Malaysia; 2 Tropical-Biological Research Unit, College of Science, University of Baghdad, Baghdad, Iraq; 3 National Diabetes Center, Mustansiriyah University, Baghdad, Iraq; 4 College of Dentistry, Uruk University, Baghdad, Iraq

**Keywords:** T2DM, Microvascular complication, IL-29, IL-41, Raftlin, ROC curve, Biomarkers, Diseases, Endocrinology, Immunology, Medical research, Nephrology

## Abstract

Type 2 diabetes mellitus (T2DM) is a dysfunctional metabolic disorder characterized by low-grade inflammation. Peripheral neuropathy (PN), nephropathy (NE), and retinopathy (RT) are prominent microvascular complications (MICs) associated with T2DM. Interleukin (IL)-29, IL-41, and raftlin are recently identified immunomodulators that are hypothesized to play a pathogenic role in T2DM, but the evidence is not conclusive. Therefore, a study was conducted to characterize serum levels of these markers in T2DM patients with and without MICs (PN, NE, and RT) and to explore their cross‑sectional associations with these MICs. In this case–control study, 154 T2DM patients were included and classified into five groups: 34 newly diagnosed patients (ND), 30 patients without MICs (DM), 30 PN patients, 30 NE patients, and 30 RT patients. In addition, a control group (HC) consisting of 60 individuals was included. Serum IL-29, IL-41, and raftlin concentrations were quantified using ELISA kits. Compared to HC, IL‑41 levels showed a consistent and significant elevation in T2DM groups, particularly in ND and NE patients, whereas IL‑29 and raftlin levels showed modest variations, most of which were not significant changes. Receiver operating characteristic curve analysis revealed that an area under the curve greater than 0.70 was recorded only for IL-41 in all T2DM patients, as well as in ND and DM patients. Logistic regression analysis demonstrated that elevated IL-41 and decreased raftlin levels were linked to significantly high and low odds ratios, respectively, in all patients, and in ND and DM patients. In contrast, no significant association was observed between MICs (PN, NE, and RT) and IL-29, IL-41, or raftlin. These findings suggest that IL-41 and raftlin may represent candidate biomarkers related to inflammatory and metabolic disturbances in T2DM and associated MICs. However, their biological and clinical relevance requires confirmation in larger longitudinal and mechanistic studies.

**Supplementary Information:**

The online version contains supplementary material available at https://doi.org/10.1038/s41598-026-55654-y.

## Introduction

Type 2 diabetes mellitus (T2DM) is a common metabolic disorder and the most prevalent type of diabetes, accounting for approximately 90% of the global burden of diabetes. Recent data raises global health concerns, with the prevalence of diabetes reaching 11.1% in 2024 and expected to rise to 13% by 2050. Furthermore, diabetes and its clinical complications account for 9.3% of global deaths. In Iraq, the age-adjusted prevalence rate of diabetes among adults (20–79 years) exceeded the global average in 2024, reaching 13.4%^1^. These data necessitate extensive studies to understand the pathophysiology of T2DM, the associated risk factors, and the accompanying clinical complications. Hyperglycemia is a hallmark of the metabolic disorder associated with T2DM. It results from a progressive loss of adequate insulin secretion from pancreatic beta-cells and is frequently associated with insulin resistance^2^. Persistent hyperglycemia and the associated pathophysiological changes can lead to the development of various microvascular complications (MICs), such as peripheral neuropathy (PN), nephropathy (NE), and retinopathy (RT), as well as macrovascular complication (MACs) such as cardiovascular and cerebrovascular diseases^3^. The etiology and pathophysiology of T2DM is complex and multifactorial. Obesity, unhealthy diet, physical inactivity, psychosocial stress, socioeconomic determinants, and environmental toxins are key risk factors that contribute to the progression of T2DM and insulin resistance in genetically predisposed individuals. This occurs through stress-induced inflammation, which ultimately leads to insulin receptor insensitivity^4^. Inflammation, particularly low-grade inflammation, is a prominent feature associated with T2DM and is considered a significant contributing factor to the risk of MICs and MACs. In fact, dysregulated production of pro-inflammatory and anti-inflammatory mediators is a functional driver of pancreatic beta-cell dysfunction, insulin resistance, and MIC damage, thereby increasing the risk of T2DM and associated clinical complications. Examples include interleukins (IL-1β, IL-4, IL-6, IL-9, IL-10, IL-13, IL-17, IL-36, IL-38, IL-39, and IL-40) and tumor necrosis factor (TNF)-α^5–9^. Recently identified immune regulators, such as IL-29, IL-41, and raftlin, have also been a focus of ongoing research into T2DM, but studies have not been well elaborated^10–12^, and a greater understanding of their role in disease progression and associated MICs is needed.

IL-29 (also known as interferon lambda 1; IFN‐λ1) was discovered in 2003 and classified as a novel cytokine belonging to the type III IFN family along with IFN‐λ2 (IL-28A), IFN‐λ3 (IL-28B), and IFN‐λ4, whose effects are functionally mediated by interaction with two receptors; IFNLR1 and IL10Rβ^13^. The potent functional effects of IL-29 in immunity have been proposed, especially considering that IL-29 is primarily expressed by epithelial cells and synthesized by important immune cells such as macrophages, dendritic cells, and T helper (h) 17 cells^14^. IL-29 has been linked to the development of a number of inflammatory autoimmune conditions, such as systemic sclerosis and arthritis, and dysregulated expression of IL-29 has been considered a risk factor in these diseases^15^. In fact, IL-29 has been proposed as one of the inflammatory cytokines and in vitro experiments have demonstrated its enhancing effects on pro-inflammatory and anti-inflammatory cytokines, such as IL-6, IL-10, IL-12, IL15, IL-23, IL-27, and TNF-α^16^. Regarding T2DM, Sardarmelli and colleagues demonstrated in vitro that Th cells in T2DM patients overproduce IL-29 compared to controls^10^. Nabiyi et al. reported that IL-29 levels, protein and gene expression, were elevated in T2DM patients who were not treated with sitagliptin (an oral hypoglycemic medication) compared to controls^17^. In Iraq, two studies reported conflicting results. In the first, serum IL-29 levels were decreased in T2DM patients compared controls^18^, while in the second, increased levels were found only in T2DM patients with RT^19^. Regarding MICs, although data are limited, a recent study has shown that IL-29 may play a role in the progression of vascular calcification, and that inhibiting this cytokine or its specific receptors may provide a new strategy for controlling vascular calcification in patients with vascular disease^20^. In addition, more recent evidence suggests that IL-29 exhibits modulating effects on angiogenesis by inhibiting endothelial cell migration associated with decreased expression of vascular endothelial growth factor^21^.

IL- 41 (also known as METRNL, cometin, and subfatin) is a novel cytokine first described in 2012 as a neurotrophic factor, homologous to the mouse Metrnl discovered in 2004. Initial studies described IL- 41 as an adipokine associated with metabolic responses, owing to its high expression in adipocytes and adipose tissue. However, subsequent investigations have demonstrated that IL- 41 is expressed in lymphoid tissues, such as the thymus and spleen, as well as in immune cells such as activated macrophages, suggesting its immunomodulatory effects on inflammation and both innate and adaptive immunity^22^. In addition, IL- 41 production is influenced by other cytokines. In this context, pro- inflammatory cytokines, such as IL- 12, IL- 17 A, and TNF- α, stimulate bone marrow macrophages to produce IL- 41, whereas regulatory cytokines, such as transforming growth factor (TGF)-β and IFN- γ, have inhibitory effects. IL- 41 has also been shown to regulate the production of chemokines and cytokines by macrophages, and mice deficient in the gene encoding IL- 41 showed defects in cytokine synthesis^23^. Inflammatory and autoimmune diseases associated with cytokine dysregulation, such as inflammatory bowel disease, psoriatic arthritis, and rheumatoid arthritis, confirm the immunomodulatory effects of IL- 41, which exhibits markedly altered levels in these diseases^24,25^. Since IL-41 is an adipokine associated with metabolic responses, there is experimental evidence in mice suggesting that this cytokine is associated with insulin sensitivity. Overexpression of IL- 41 in adipose tissue has been linked to effects that counteract insulin resistance, indicating a role for IL- 41 in T2DM pathogenesis and its associated MICs^22^. In addition, IL- 41 has been shown to affect reactive oxygen species levels and control activation of the inflammasome component NLRP3, which is believed to play an influential role in T2DM and related vascular complications^26^. Regarding human studies, the results have been inconsistent, with higher and lower levels of IL- 41 reported in T 2 DM patients compared with controls. In addition, IL- 41’ s role in the pathogenesis of MICs associated with T 2 DM has not been well investigated.

Raftlin (also known as lipid raft linker 1; RFTN-1) was described in 2003 as a novel, vital, and essential component of the lipid raft proteins in Raji B cells. Lipid rafts are cell membrane micro-domains rich in cholesterol, sphingolipids, and phospholipids, which act as signal transduction regulators to maintain cellular homeostasis. Structural proteomic analyses have shown that raftlin co-localizes with the B-cell receptor (BCR), suggesting a functional role in regulating receptor-mediated signaling^27^. Subsequent studies have gone further, indicating a role for raftlin in mediating important aspects of immune function related to B and T lymphocyte signaling pathways, as well as signaling pathways involving two Toll-like receptors (TLR3 and TLR4). Furthermore, raftlin has been associated with regulating effects on vascular inflammatory responses by influencing oxidative stress and antioxidant responses^28^. Raftlin has also been shown to indirectly promote pathogenic Th17 cells, suggesting a functional role for raftlin in inflammatory diseases mediated by these cells^29^. In fact, recent studies have indicated that raftlin shows dysregulated expression in some inflammatory and autoimmune conditions, such as arthritis and otosclerosis^30,31^. In T2DM, a literature review revealed only one study that determined serum raftlin levels in two patient groups; with and without RT. Raftlin levels were significantly lower in all T2DM patients versus controls, while no significant variation was observed between patients with and without RT^12^.

As described above, IL-29, IL-41, and raftlin represent important research targets for understanding the pathogenesis of T2DM, particularly disease-associated MICs. However, previous studies investigating IL-29, IL-41, and raftlin in T2DM primarily compared patients and controls without identifying MICs, or focused on a single MIC or marker, so a comprehensive comparison across multiple MICs is lacking. Therefore, this study primarily aimed to investigate whether serum levels of IL-29, IL-41, and raftlin differ between T2DM patients with and without MICs, and to assess their association with specific complications such as PN, NE, and RT. As a secondary objective, the comparisons were expanded to include newly diagnosed (ND) patients, and potential confounding factors such as age, gender, body mass index (BMI), disease duration, and medications were evaluated.

## Materials and methods

### Study populations

This case–control study was approved by the Ethics Committee of the College of Science (University of Baghdad) and Ibn Al-Haitham Eye Teaching Hospital (approval numbers: CSEC/1223/0137 and 9101, respectively). The study protocol was performed in accordance with the relevant guidelines and regulations, including the institutional, national, and international standards for research involving human participants. All participants provided informed consent. Participants included 154 patients with T2DM (34 ND, 30 DM [patients without MICs], 30 PN, 30 NE, and 30 RT) and 60 healthy controls (HC). Patients were referred to the outpatient clinics at the National Center for Diabetes (Mustansiriyah University; ND, DM, PN, and NE patients) and Ibn Al-Haitham Eye Teaching Hospital (Ministry of Health; RT patients) in Baghdad from July 2024 to February 2025. All T2DM patients met the American Diabetes Association (ADA) diagnostic criteria^32^. Inclusion criteria were: age ≥ 18 years, both genders, MIC (PN, NE, or RT), ND (not receiving hypoglycemic medications), and DM (absence of MICs). Exclusion criteria were: T1DM, gestational diabetes, MAC, inflammatory or autoimmune disorder, acute or chronic infection, hepatic dysfunction or chronic liver disease (viral hepatitis, cirrhosis, clinically documented fatty liver disease/steatohepatitis, or unexplained elevation of liver transaminases), end-stage renal disease or dialysis, malignancy, current use of medications known to affect immune status (such as corticosteroids), incomplete clinical data, and lack of informed consent. The HC group consisted of non-diabetic healthy individuals, selected from among blood donors and healthcare workers. To exclude prediabetes and diabetes, individuals with a fasting plasma glucose level ≥ 100 mg/dL and/or a glycated hemoglobin (HbA1c) level ≥ 39 mmol/mol (5.7%) were not included, according to the Standards of Care in Diabetes-2024^32^.

### Baseline data

A structured case report form was designed to record demographic and clinical data for each patient (age, gender, weight, height, and age of T2DM onset). In addition, T2DM duration, family history of diabetes (sib, parent, aunt, uncle, grandmother, or grandfather), and current treatment regimen (oral hypoglycemic medications or insulin therapy) were obtained through direct interviews with patients and review of medical records. BMI was calculated by dividing body weight (in kilograms; kg) by the square of the height (in meters; m). Participants were classified into two BMI groups: normal weight (NW; BMI = 18.5–24.9 kg/m^2^) and overweight/obese (O/O; BMI ≥ 25.0 kg/m^2^)^33^.

### Biochemical parameters

Between 8:00 a.m. and 10:00 a.m., a blood sample was drawn into a polymer gel separator tube after an overnight fast of approximately 10–12 h. Blood was allowed to clot at room temperature (20–25 °C) for approximately 20 min, and then centrifuged (3000 rpm; 10 min). The obtained serum was aliquoted into labelled cryovials and kept at − 20 °C until analysis. Serum levels of the following biochemical parameters were quantified: FPG, total cholesterol (TC), triglycerides (TG), high-density lipoprotein (HDL), low-density lipoprotein (LDL), very-low-density lipoprotein (VLDL), serum creatinine (sCR), and blood urea nitrogen (BUN). A further blood sample was drawn into an EDTA tube and used to determine HbA1c level. These analyses were performed using an automated biochemical analyzer (Roche/Hitachi Cobas c311, Germany). In addition, a morning urine sample was also obtained and tested to determine the levels of microalbumin (uALB) and CR (uCR) (DCA Vantage analyzer; Fisher Scientific, USA), which were used to calculate the urine albumin-to-creatinine ratio (uACR)^34^.

### Diagnosis of MICs

Three MICs were investigated; PN, NE, and RT. PN was diagnosed using the Toronto Clinical Neuropathy Score (TCNS), which measures the severity of neuropathy on a scale from 0 to 19, combining three components: symptom scores, reflex scores, and physical examination findings. The total TCNS was calculated by summing these components, providing a standardized measure of PN severity as follows: 0–5, 6–8, 9–11, and ≥ 12 TCNS ranges indicate no, mild, moderate, and severe PN, respectively^35^. We did not consider this classification of PN severity due to small sample size, and the study included any patient with TCNS ≥ 6. Diabetic NE was defined as a moderate increase in albuminuria, based on uACR range ≥ 3 to < 30 mg/mmol, according to the KDIGO 2024 classification of albuminuria A2^36^. sCR was measured and the estimated glomerular filtration rate was taken into account in the clinical assessment to exclude advanced kidney disease, end-stage renal failure, or dialysis. RT was diagnosed by an ophthalmologist based on the detection of characteristic retinal changes, including retinal photocoagulation scars, cotton wool spots, microaneurysms, and retinal hemorrhages, using slit-lamp biomicroscopy^37^.

### IL-29, IL-41, and raftlin immunoassays

Serum IL-29, IL-41, and raftlin concentrations were measured using ELISA test kits (catalogue number: ELK1784, ELK7431, and ELK9287, respectively) following manufacturer’s protocols (ELK Biotechnology Company, China; https://www.elkbiotech.com). The detection range (sensitivity) for these kits was: 7.82–500 pg/mL for IL-29 (2.6 pg/mL), 1.57–100 ng/mL for IL-41 (0.66 ng/mL), and 78.13–5000 pg/mL for raftlin (31.1 pg/mL). Kits were based on similar sandwich ELISA testing principles. Briefly, the supplied microtiter plate (96 wells) was pre-coated with an antibody specific to human IL-29, IL-41, or raftlin. Standards (0, 7.82, 15.63, 31.25, 62.5, 125, 250, and 500 pg/mL for IL-29; 0, 1.57, 3.13, 6.25, 12.5, 25, 50, and 100 ng/mL for IL-41; and 0, 78.13, 156.25, 312.5, 625, 1250, 2500, and 5000 pg/mL for raftlin) or serum samples were pipetted into designated microplate wells, and a biotin-conjugated antibody specific for human IL-29, IL-41, or raftlin was added. Horseradish peroxidase-conjugated avidin was then pipetted into each microplate well and incubated. Substrate solution was then added, and after development of blue color, stop solution was added, resulting in the development of yellow color. The color intensity is proportional to concentration, which was monitored using a microplate reader (HumaReader HS; Human Diagnostics; Germany) to measure optical density at 450nm wavelength. IL-29, IL-41, and raftlin concentrations were determined using generated standard curves, and assay reliability was ensured using estimated R^2^, which was 0.993, 0.989, and 0.999, respectively.

### Statistical analysis

Baseline clinical and laboratory data were presented either as numbers and percentages for categorical variables or as mean and standard deviation (SD) for quantitative variables, and Pearson’s chi-square test or one-way analysis of variance (ANOVA) test was used to identify significant differences, respectively. Shapiro–Wilk and Kolmogorov–Smirnov tests (tests for normality) showed that IL-29, IL-41, and raftlin concentrations did not conform to a normal distribution. Therefore, median and interquartile range (IQR: 25–75%) were used to present these concentrations, and Mann–Whitney U test was used to assess significant differences. The potential of these serum markers to differentiate between patients and HC was assessed using receiver operating characteristic (ROC) curve analysis. Based on this analysis, the area under the curve (AUC) and 95% confidence interval (CI) were calculated. Multinomial logistic regression analysis was conducted to analyze the association between IL-29, IL-41, and raftlin (predictors) and T2DM or its MICs. Odds ratio (OR) and 95% CI were estimated through this analysis. The bivariate correlation coefficient (r_s_) between variables was calculated using Spearman’s rank correlation test. Statistical significance was set at a two-tailed probability (*p*) of less than 0.05. The *p*-value was corrected (*pc*) for multiple comparisons using Dunn’s test or Bonferroni method as appropriate. GraphPad Prism version 9.4.1 (San Diego, California USA) and IBM SPSS Statistics 25.0 (Armonk, NY: IBM Corp.) were used to accomplish statistical analyses.

A *post-hoc* analysis was performed to estimate sample size power under Wilcoxon-Mann-Whiney test using G*power (version 3.1.9.7), which was programmed using the following inputs: 154 T2DM patients, 60 HC, 0.05 two-tailed α error *p*, and 0.5 effect size d. The power of sample size was 0.86; suggesting that the included number of patients and HC was statistically suitable to validate obtained results^38^. The estimated sample size power was based on all patients; however, classifying patients into subgroups, particularly those with MICs (PN, NE, and RT; 30 patients per subgroup), may weaken the sample size power, and larger patient groups are recommended. Therefore, the results of ROC curve analysis and logistic regression analysis should be interpreted with caution.

## Results

### Baseline data

Age (mean ± SD) was similar in ND patients (46 ± 13 years) and HC (46 ± 9 years), but increased in DM, PN, NE, and RT patients (48 ± 9, 54 ± 9, 50 ± 10, and 58 ± 9 years, respectively), and overall comparison indicated a significant difference (*p* < 0.001). Age group frequencies also varied significantly among the groups of patients and HC (*p* < 0.001), with over 70% of PN and RT patients being classified in the 51–68 year age group, while over 60% of ND, DM, and NE patients, as well as HC, were classified in the 18–50 year age group. Male and female frequencies in T2DM groups (ND, DM, PN, NE, and RT) and HC showed a non-significant variation (*p* = 0.063). Similarly, BMI showed a non-significant difference as most patients and HC were O/O with a BMI greater than 25 kg/m^2^ (*p* = 0.703). Family history of diabetes (sib, parent, aunt, uncle, grandmother, or grandfather) was found in 58.8, 60.0, 56.7, 63.3, and 80.0% of ND, DM, PN, NE, and RT patients, respectively. Interestingly, 43.3% of HC also showed a family history of diabetes. Comparison between patients and HC showed a significant difference (*p* = 0.039), which could be ascribed to the apparent higher frequency of family history in RT patients (80.0%). Regarding medication, 53.3, 80.0, and 50.0% of PN, NE, and RT patients, respectively, were receiving oral hypoglycemic therapy, while 76.7% of DM patients were receiving insulin therapy. HbA1c and FPG levels were above normal in the five groups of T2DM patients (range: 64–80 mmol/mol and 208–270 mg/dL, respectively), while normal levels were recorded in HC (30 ± 5 mmol/mol and 89 ± 9 mg/dL, respectively), indicating that HC individuals were not diabetic. Kidney function parameters (BUN and sCR) and lipid profile (TC, TG, HDL, LDL, and VLDL) were significantly different between T2DM patients and HC. Regarding uACR, it exhibited significantly higher levels in NE and RT patients compared to ND, MD, and PN patients (7.8 ± 3.6 and 5.5 ± 5.9 vs. 2.6 ± 2.1, 1.5 ± 0.5, and 2.8 ± 3.2 mg/mmol, respectively; *p* < 0.001) (Table 1). Interestingly, 16 RT patients (53.3%) had uACR ≥ 3 mg/mmol, indicating microalbuminuria (nephritis; RT + NE), while 14 patients (46.7%) showed uACR < 3 mg/mmol (without nephritis; RT-NE) (data not shown).

**Table 1:** Baseline data of patients with type 2 diabetes mellitus and non-diabetic healthy controls.

Parameter	T2DM patients; n = 154					HC; n = 60	*p* value
	ND; n = 34	DM; n = 30	PN; n = 30	NE; n = 30	RT; n = 30		
Age; year	46 ± 13	48 ± 9	54 ± 9	50 ± 10	58 ± 9	46 ± 9	** < 0.001**
AAO; year	46 ± 13	44 ± 10	42 ± 9	44 ± 9	42 ± 9	NA	0.548
DD; year	< 3 months	4 ± 2	12 ± 5	6 ± 5	15 ± 5	NA	** < 0.001**
BMI; kg/m^2^	30.9 ± 5.0	31.2 ± 4.2	29.6 ± 4.5	30.5 ± 5.0	29.1 ± 4.6	29.9 ± 5.4	0.516
HbA1c; mmol/mol	69 ± 18	64 ± 17	80 ± 19	75 ± 17	75 ± 16	30 ± 5	** < 0.001**
FPG; mg/dL	211 ± 84	208 ± 84	270 ± 117	226 ± 88	244 ± 97	89 ± 9	** < 0.001**
uALB; mg/L	38 ± 40	17 ± 10	33 ± 42	128 ± 46	69 ± 63	NA	** < 0.001**
uCR; mmol/L	14.7 ± 7.8	12.7 ± 7.5	11.6 ± 7.0	17.6 ± 7.2	14.4 ± 8.2	NA	**0.029**
uACR; mg/mmol	2.6 ± 2.1	1.5 ± 0.5	2.8 ± 3.2	7.8 ± 3.6	5.5 ± 5.9	NA	** < 0.001**
BUN; mg/dL	24 ± 8	26 ± 8	28 ± 6	29 ± 11	41 ± 16	24 ± 6	** < 0.001**
sCR; mg/dL	0.6 ± 0.1	0.7 ± 0.1	0.7 ± 0.2	0.7 ± 0.2	0.9 ± 0.3	0.7 ± 0.1	** < 0.001**
TC; mg/dL	216 ± 46	188 ± 36	198 ± 41	199 ± 52	197 ± 57	175 ± 33	**0.001**
TG; mg/dL	187 ± 105	172 ± 80	183 ± 91	199 ± 136	149 ± 72	129 ± 58	**0.003**
HDL; mg/dL	41 ± 7	46 ± 16	46 ± 13	44 ± 12	45 ± 8	38 ± 10	**0.003**
LDL; mg/dL	110 ± 47	89 ± 37	103 ± 31	103 ± 44	80 ± 52	106 ± 27	**0.014**
VLDL; mg/dL	54 ± 29	36 ± 18	38 ± 20	43 ± 29	34 ± 16	25 ± 12	** < 0.001**
Age; year							
18–50	21 (61.8)	19 (63.3)	8 (26.7)	19 (63.3)	2 (6.7)	36 (60.0)	** < 0.001**
51–68	13 (38.2)	11 (36.7)	22 (73.3)	11 (36.7)	28 (93.3)	24 (40.0)	
Gender							
Male	16 (47.1)	16 (53.3)	19 (63.3)	24 (80.0)	18 (60.0)	29 (48.3)	0.063
Female	18 (52.9)	14 (46.7)	11 (36.7)	6 (20.0)	12 (40.0)	31 (51.7)	
BMI							
NW	5 (14.7)	2 (6.7)	6 (20.0)	3 (10.0)	3 (10.0)	8 (13.3)	0.703
O/O	29 (85.3)	28 (93.3)	24 (80.0)	27 (90.0)	27 (90.0)	52 (86.7)	
Family history							
Yes	20 (58.8)	18 (60.0)	17 (56.7)	19 (63.3)	24 (80.0)	26 (43.3)	**0.039**
No	14 (41.2)	12 (40.0)	13 (43.3)	11 (36.7)	6 (20.0)	34 (56.7)	
Medication							
Oral	NA	7 (23.3)	16 (53.3)	24 (80.0)	15 (50.0)	NA	** < 0.001**
Insuline	NA	23 (76.7)	14 (46.7)	6 (20.0)	15 (50.0)	NA	

T2DM: type 2 diabetes mellitus; ND: newly diagnosed patients; DM: patients without diabetes-related microvascular complications; PN: patients with peripheral neuropathy; NE: patients with nephropathy; RT: patients with retinopathy; HC: non-diabetic healthy controls; AAO: age at onset; DD: disease duration; BMI: body mass index; NW: normal weight; O/O: overweight/obese; HbA1c: Glycated hemoglobin; FPG: fasting plasma glucose; uALB: urine albumin; uCR: urine creatinine; uACR: urine albumin-to-creatinine ratio; BUN: blood urea nitrogen; sCR: serum creatinine; TC: total cholesterol; TG: triglycerides; HDL: high-density lipoprotein; LDL: low-density lipoprotein; VLDL: very-low-density lipoprotein; NA: not applicable; *p*: Two-tailed probability (significant *p* value is indicated in bold). Scale variables are given as mean ± standard deviation, and significance was determined using one-way analysis of variance (ANOVA) test. Categorical variables are given as absolute number followed by percentage frequency in parentheses, and significance was determined using Pearson chi-square test.

### Serum IL-29, IL-41, and raftlin concentrations

**Fig. 1:**
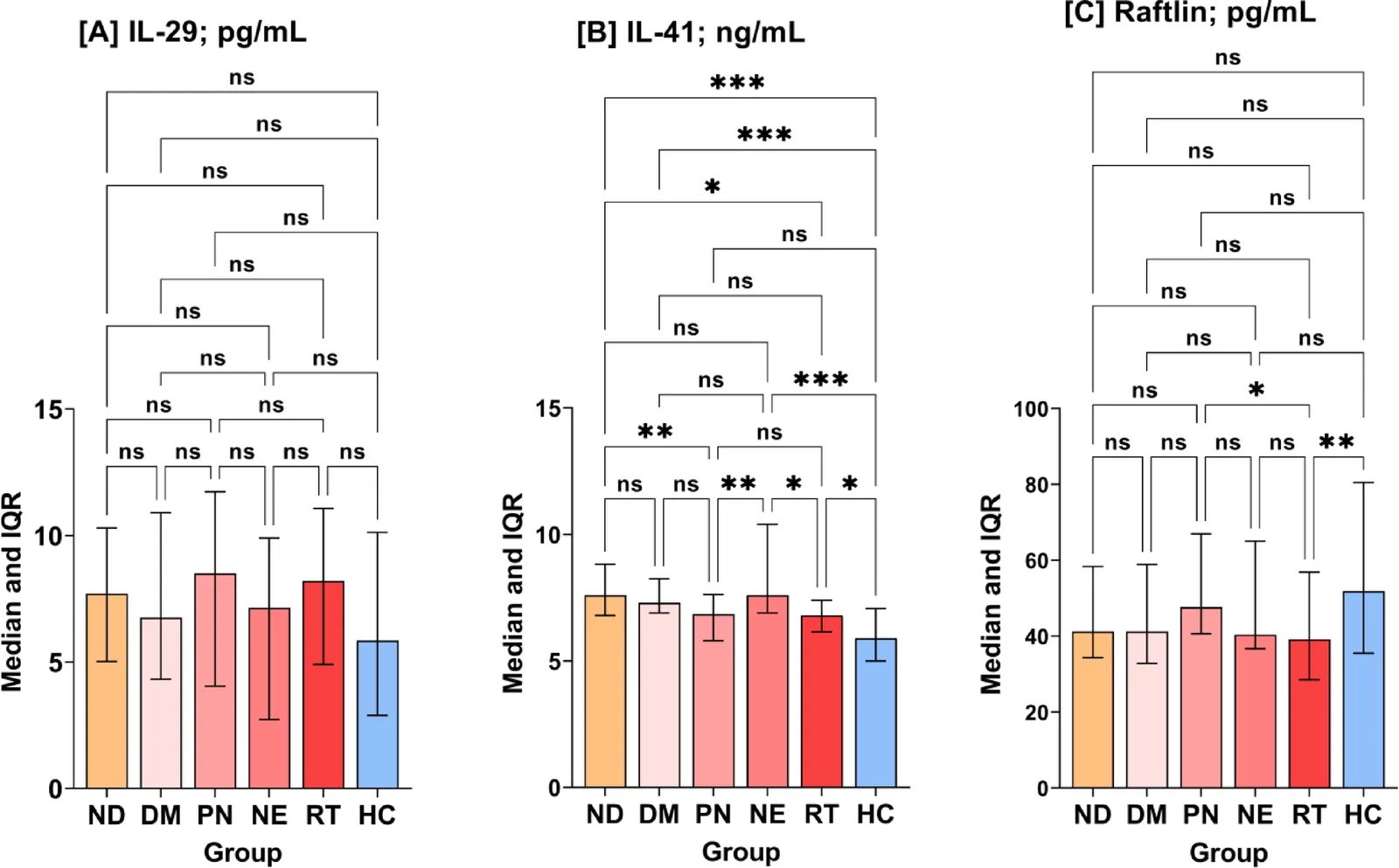
Column-bar plots showing serum levels of interleukin (IL)-29 (plot **A**), IL-41 (plot **B**), and raftlin (plot **C**) in patients with type 2 diabetes mellitus (ND: Newly diagnosed patients [n = 34]; DM: Patients without diabetes-related microvascular complications [n = 30]; PN: Patients with peripheral neuropathy [n = 30]; NE: Patients with nephropathy [n = 30]; RT: Patients with retinopathy [n = 30 patients]) and non-diabetic healthy controls (HC [n = 60]). Columns indicate median, while bars indicate interquartile range (IQR: 25–75%). Significance was determined using Mann–Whitney U test. Statistically significant probabilities are indicated by asterisks (*p < 0.05; **p < 0.01; ***p < 0.001; ns: not significant [*p* > 0.05]). IL-29 levels showed no significant differences between the six groups studied. IL-41 levels were significantly higher in ND (*p* < 0.001), DM (*p* < 0.001), NE (*p* < 0.001), and RT (*p* = 0.034) patients compared HC. IL-41 levels were also significantly different between some groups of patients: ND versus PN (*p* = 0.006), ND versus RT (*p* = 0.012), PN versus NE (*p* = 0.007), and NE versus RT (*p* = 0.013). Raftlin levels decreased significantly in RT patients compared to PN patients or HC (*p* = 0.045 and 0.009, respectively). However, when the *p* value was corrected for multiple comparisons using Dunn’s test, only elevated IL-41 levels in ND, DM, and NE patients maintained a significant difference (*pc* < 0.001) compared to HC.

IL-29 concentrations (median [IQR]) were higher in the five groups of T2DM patients compared to HC, particularly PN and RT patients (8.5 [4.1–11.7] and 8.2 [4.9–11.1] vs. 5.9 [2.9–10.1] pg/mL, respectively), but no significant differences were recorded (*p* = 0.137 and 0.182, respectively). In addition, IL-29 concentrations did not exhibit significant variation between ND, DM, PN, NE, and RT patients (*p* > 0.05) (Fig. 1A). IL-41 concentrations were significantly higher in ND (7.6 [6.8–8.8] ng/mL; *p* < 0.001), DM (7.3 [6.9–8.3] ng/mL; *p* < 0.001), NE (7.6 [6.9–10.4] ng/mL; *p* < 0.001), and RT (6.8 [6.2–7.4] ng/mL; *p* = 0.034) patients compared HC (5.9 [5.0–7.1] ng/mL). PN patients also showed higher concentrations compared to HC, but the difference was not significant (6.9 [5.8–7.6] vs. 5.9 [5.0–7.1] ng/mL; *p* = 0.065). IL-41 concentrations were significantly different between some groups of patients: ND compared to PN (7.6 [6.8–8.8] vs. 6.9 [5.8–7.6] ng/mL; *p* = 0.006), ND compared to RT (7.6 [6.8–8.8] vs. 6.8 [6.2–7.4] ng/mL; *p* = 0.012), PN compared to NE (6.9 [5.8–7.6] vs. 7.6 [6.9–10.4] ng/mL; *p* = 0.007), and NE compared to RT (7.6 [6.9–10.4] vs. 6.8 [6.2–7.4] ng/mL; *p* = 0.013) (Fig. 1B). Raftlin tend to show decreased concentrations in the five groups of T2DM patients compared to HC but without a significant difference (*p* = 0.083 for overall comparison). The greatest decrease in raftlin concentrations occurred in RT patients and it was significant compared to PN patients and HC (39.1 [28.6–56.9] vs. 47.7 [40.6–66.9] and 51.9 [35.5–80.5] pg/mL; *p* = 0.045 and 0.009, respectively) (Fig. 1C). However, when the *p*-value was corrected for multiple comparisons using Dunn’s test (IL-41 and raftlin), only elevated IL-41 levels in ND, DM, and NE patients maintained a significant difference (*pc* < 0.001) compared to HC.

**Fig. 2:**
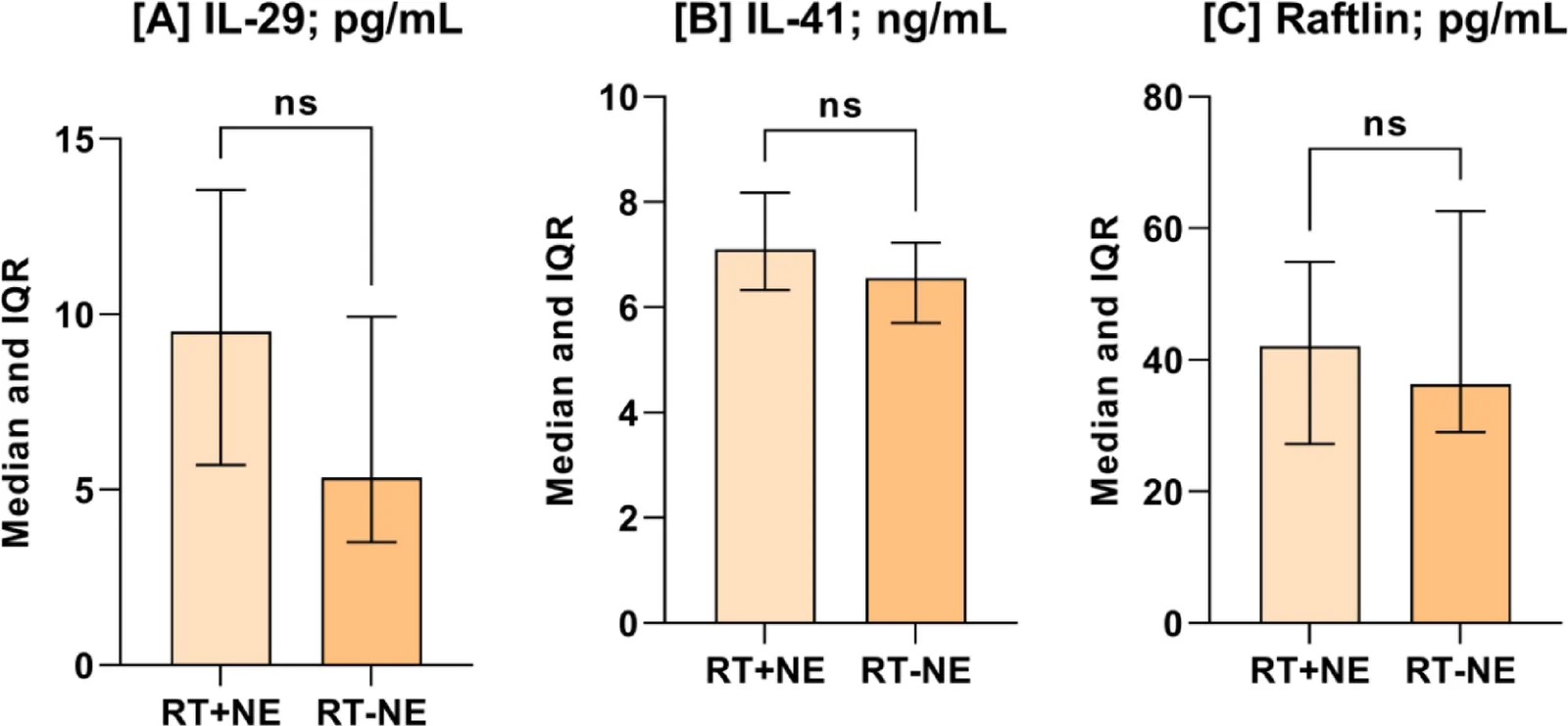
Column-bar plots showing serum levels of interleukin (IL)-29 (plot **A**), IL-41 (plot **B**), and raftlin (plot **C**) in type 2 diabetes mellitus patients with retinopathy (RT) classified by nephritis (RT patients with nephritis; RT + NE [n = 16] and RT patients without nephritis; RT-NE (n = 14]). Columns indicate median, while bars indicate interquartile range (IQR: 25–75%). Significance was determined using Mann–Whitney U test (ns: not significant [*p* > 0.05]). IL-29, IL-41, and raftlin levels were higher in RT + NE patients compared to RT-NE, but without significant differences (*p* = 0.133, 0.176, and 0.878, respectively).

Since NE was overlapping among RT patients and 53.3% of them had NE (RT + NE) and 46.7% did not have NE (RT-NE), this overlap may affect the levels of IL-29, IL-41, and raftlin in this group of T2DM patients. In fact, IL-29 (9.5 [5.7–13.6] vs. 5.4 [3.5–9.9] pg/mL), IL-41 (7.1 [6.3–8.2] vs. 6.6 [5.7–7.2] ng/mL), and raftlin (42.1 [27.3–54.9] vs. 36.3 [29.1–62.6] pg/mL) levels were elevated in RT + NE patients compared to RT-NE, but without significant differences (*p* = 0.133, 0.176, and 0.878, respectively) (Fig. 2).

### ROC curve analysis

**Fig. 3:**
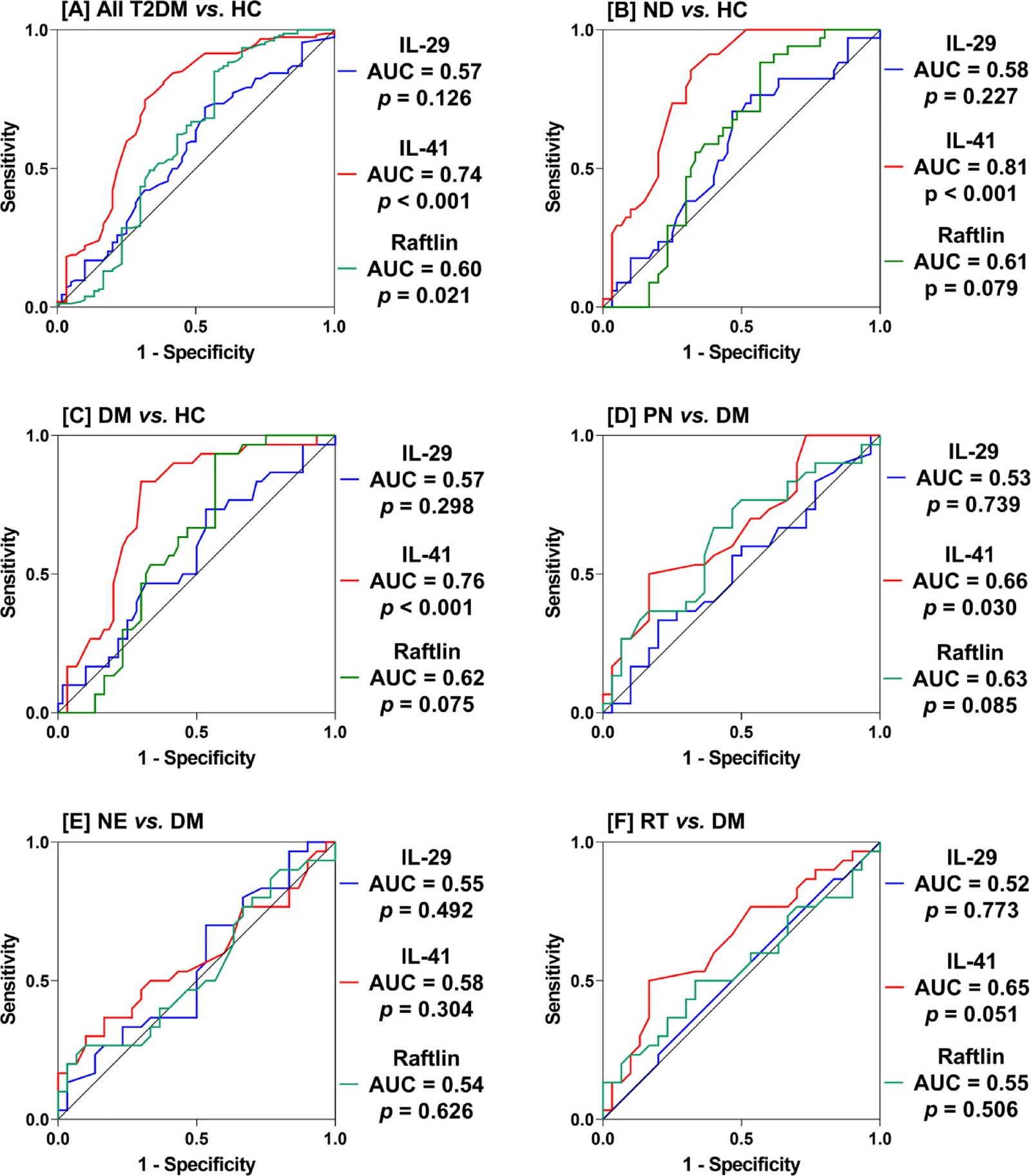
Receiver operating characteristic (ROC) curve analysis of interleukin (IL)-29, IL-41, and raftlin in all type 2 diabetes mellitus patients (T2DM; n = 154; plot **A**), newly diagnosed patients (ND; n = 34; plot **B**), patients without diabetes-related microvascular complications (DM; n = 30; plot **C**), patients with peripheral neuropathy (PN; n = 30; plot D), patients with nephropathy (NE; n = 30; plot **E**), and patients with retinopathy (RT; n = 30; plot **F**), For all T2DM patients, as well as ND and DM patients, ROC curve analysis was conducted versus non-diabetic healthy controls (n = 60). For PN, NE, and RT patients, ROC curve analysis was conducted versus DM patients. ROC curve analysis is used to evaluate the performance of a test variable in distinguishing between healthy and diseased populations by estimating area under the curve (AUC). An AUC range between 0.50 and 0.59 indicates no discrimination ability, while an AUC range between 0.60 and 0.69, 0.70 and 0.79, 0.80 and 0.89, and 0.90 and 1.0 indicates poor, acceptable, excellent, and outstanding discrimination ability, respectively^39^. The AUC value and the two-tailed probability (*p*) are shown. An AUC value > 0.70 was recorded for only IL-41 in all T2DM patients, and ND and DM patients (AUC = 0.74, 0.81 and 0.76, respectively).

The potential use of IL-29, IL-41, and raftlin as markers that could be used to differentiate between T2DM patients without MICs (ND and DM groups) and HC, or patients with MICs (PN, NE, and RT groups) and patients without MICs (DM group), was assessed by estimating the AUC value through ROC curve analysis. An AUC range between 0.5 and 0.59 indicates no discrimination ability, while an AUC range between 0.6 and 0.69, 0.7 and 0.79, 0.8 and 0.89, and 0.9 and 1.0 indicates poor, acceptable, excellent, and outstanding discrimination ability, respectively^39^. An AUC value greater than 0.70 was estimated only for IL-41 in all T2DM patients, as well as in ND and DM patients (AUC = 0.74, 0.81 and 0.76, respectively); an observation that may indicate IL-41’s ability to distinguish between these groups of patients and HC, particularly ND cases. With regard to MICs (PN, NE, and RT), the results of ROC analysis did not support the ability of any of the studied markers (IL-29, IL-41, and raftlin) to distinguish between patients with these MICs and those without MICs, as evidenced by an AUC value of less than 0.70 (Fig. 3).

### Logistic regression analysis

Initially, logistic regression analysis was performed to evaluate the association of IL-29, IL-41, and raftlin with T2DM in all patients, as well as in ND and DM groups, after adjusting the analysis for age, gender, and BMI (HC was the reference group). The analysis was then re-conducted on patients with MICs (PN, NE, and RT), with DM considered as a reference group (patients without MICs), and the analysis was adjusted for age, gender, medications, disease duration, BMI, HbA1c, BUN, and sCR. Elevated IL-41 levels were significantly associated with T2DM, whether the analysis was conducted on all patients (OR = 1.61; 95% CI = 1.29–2.02; *pc* = 0.002) or on ND (OR = 2.05; 95% CI = 1.52–2.76; *pc* = 0.001) and DM (OR = 1.80; 95% CI = 1.32–2.45; *pc* = 0.001) groups. However, the strongest association was recorded in ND group (OR = 2.05). Low levels of raftlin also showed a significant association with T2DM, but the *pc*-value remained significant only in all patients (OR = 0.98; 95% CI = 0.97–0.99; *pc* = 0.009). IL-29 did not show a significant association with T2DM in all patients, in ND patients, or in DM patients. Regarding PN, NE, and RT, IL-29, IL-41, and raftlin demonstrated no significant association in patients with these MICs versus DM patients (Table 2).

**Table 2:** Multinomial logistic regression model after adjusting for age, gender, and bod mass index to analyze the association between IL-29, IL-41, and raftlin (predictors) and the likelihood of developing type 2 diabetes mellitus and its microvascular complications.

T2DM group	Reference category	Predictor	B	SE	Wald	DF	*p* value (*pc*)	Exp (B)	95% CI	
									LB	UP
All patients^†^; n = 154	HC; n = 60	IL-29	0.06	0.03	2.86	1	0.091	1.06	0.99	1.13
		IL-41	0.48	0.12	17.08	1	** < 0.001 (0.002)**	1.61	1.29	2.02
		Raftlin	− 0.02	0.01	8.88	1	**0.003 (0.009)**	0.98	0.97	0.99
ND^†^; n = 34	HC; n = 60	IL-29	0.04	0.04	0.88	1	0.348	1.04	0.96	1.14
		IL-41	0.72	0.15	21.88	1	** < 0.001 (0.001)**	2.05	1.52	2.76
		Raftlin	− 0.02	0.01	5.01	1	**0.025** (0.075)	0.98	0.96	1.00
DM^†^; n = 30	HC; n = 60	IL-29	0.06	0.05	1.96	1	0.161	1.07	0.98	1.17
		IL-41	0.59	0.16	13.81	1	** < 0.001 (0.001)**	1.80	1.32	2.45
		Raftlin	− 0.02	0.01	5.47	1	**0.019** (0.057)	0.98	0.96	1.00
PN^‡^; n = 30	DM; n = 30	IL-29	0.11	0.08	2.15	1	0.143	1.12	0.96	1.31
		IL-41	− 0.44	0.28	2.53	1	0.112	0.64	0.37	1.11
		Raftlin	0.03	0.02	1.43	1	0.231	1.03	0.98	1.08
NE^‡^; n = 30	DM; n = 30	IL-29	0.01	0.08	0.03	1	0.871	1.01	0.87	1.18
		IL-41	0.17	0.23	0.55	1	0.458	1.18	0.76	1.85
		Raftlin	0.02	0.02	0.73	1	0.394	1.02	0.98	1.06
RT^‡^; n = 30	DM; n = 30	IL-29	0.11	0.08	1.71	1	0.191	1.12	0.95	1.32
		IL-41	− 0.22	0.30	0.53	1	0.466	0.81	0.45	1.44
		Raftlin	0.003	0.03	0.01	1	0.913	1.00	0.95	1.06

T2DM: type 2 diabetes mellitus; ND: newly diagnosed patients; DM: patients without diabetes-related microvascular complications; PN: patients with peripheral neuropathy; NE: patients with nephropathy; RT: patients with retinopathy; IL: interleukin; B: multinomial logistic regression coefficient; SE: standard error; Wald: wald chi-square test; DF: degrees of freedom; *p*: probability; *pc*: corrected probability for multiple comparisons (significant *p* value is indicated in bold); Exp(B): Exponentiation of the coefficient (odds ratios for the predictors); CI: confidence interval; LB: lower bound; UB: upper bound; ^†^analysis was adjusted for age, gender, and body mass index (BMI); ^‡^analysis was adjusted for age, gender, BMI, disease duration, medication, glycated hemoglobin, blood urea nitrogen, and serum creatinine.

### Impact of T2DM characteristics on IL-29, IL-41, and raftlin concentrations

Serum concentrations of IL-29, IL-41, and raftlin were stratified according to age group (18–50 vs. 51–68 years), gender (male vs. female), BMI (NW vs. O/O), family history of diabetes (yes vs. no), and medication (oral hypoglycemic therapy vs. insulin therapy) in the five groups of T2DM (ND, DM, PN, NE, and RT). There were no significant differences in these strata, except for raftlin in the classification of family history in PN patients. Raftlin concentrations (median [IQR]) were significantly lower in PN patients with a family history of diabetes compared to PN patients without a family history of diabetes (46.4 [38.0–50.2] vs. 61.4 [48.3–69.9] pg/mL; *p* = 0.040) (Supplementary Table S1).

### Spearman’s rank correlation analysis

**Fig. 4:**
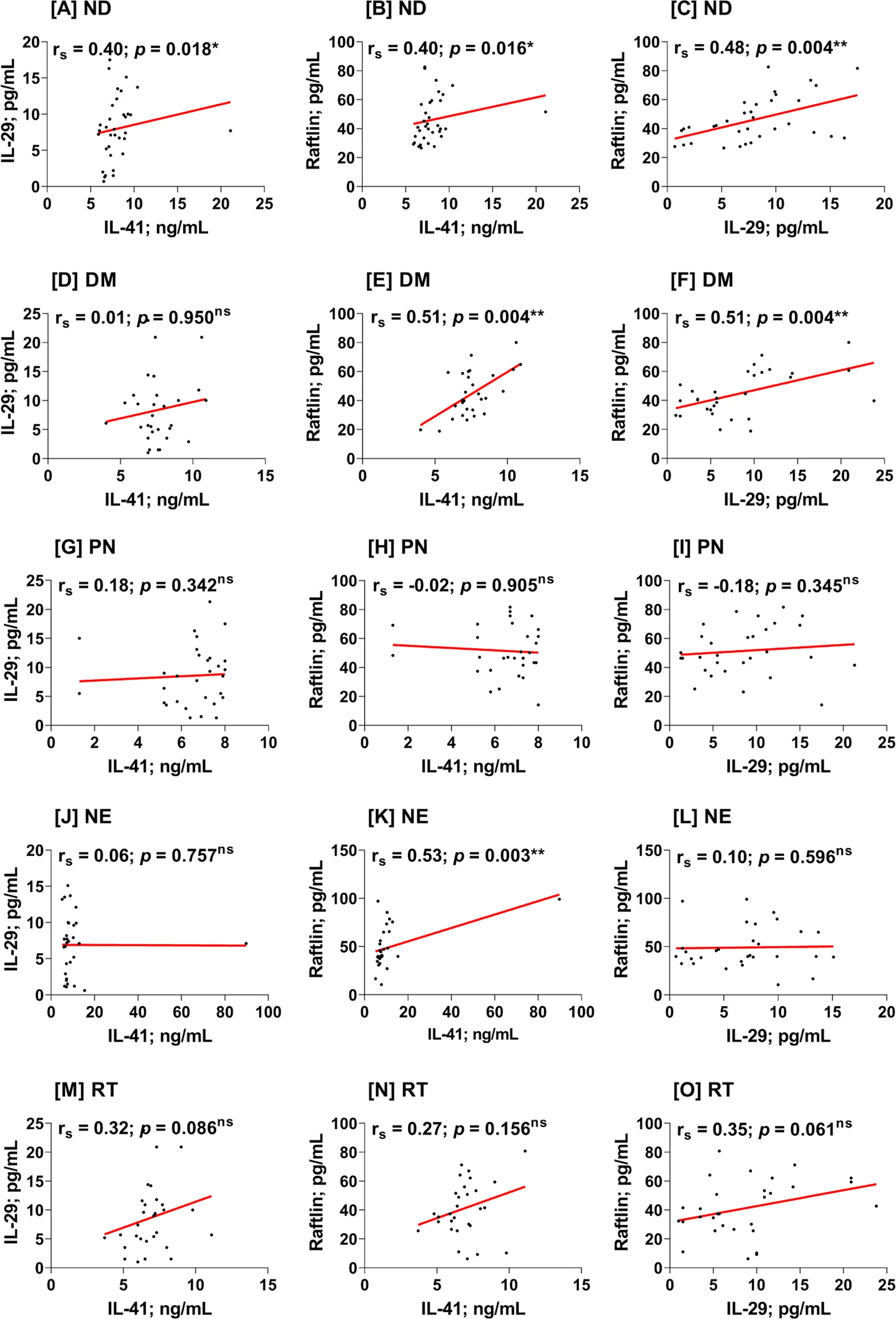
Spearman’s rank correlation analysis between interleukin (IL)-29, IL-41, and raftlin in patients with type 2 diabetes mellitus (ND: Newly diagnosed patients [n = 34; plots **A**, **B**, and **C**]; DM: Patients without diabetes-related microvascular complications [n = 30; plots **D**, **E**, and **F**]; PN: Patients with peripheral neuropathy [n = 30; plots **G**, **H**, and **I**]; NE: Patients with nephropathy [n = 30; plots **J**, **K**, and **L**]; RT: Patients with retinopathy [n = 30; plots **M**, **N**, and **O**]). As indicated by the correlation coefficient (r_s_) and two-tailed probability (*p*), the correlation between IL-29, IL-41, and raftlin showed group-dependent patterns.

Bivariate correlation analysis between IL-29, IL-41, and raftlin in the five groups of T2DM patients showed that the correlation pattern depends on the group being studied. In ND patients, significant positive correlations included: IL-41-IL-29 (r_s_ = 0.40; *p* = 0.018), IL-41-raftlin (r_s_ = 0.40; *p* = 0.016), and IL-29-raftlin (r_s_ = 0.48; *p* = 0.004). In DM patients, significant positive correlations included: IL-29-raftlin (r_s_ = 0.51; *p* = 0.004) and IL-41-raftlin (r_s_ = 0.51; *p* = 0.004). In NE patients, only IL-41 and raftlin showed a significant positive correlation (r_s_ = 0.53; *p* = 0.003). In PN and RT patients, there were no significant correlations (Fig. 4). The correlation analysis was also conducted between IL-29, IL-41, and raftlin and clinical and laboratory variables in the five groups of T2DM patients. Only NE and RT patients showed significant correlations. In NE patients, IL-41 showed a positive correlation with BMI (r_s_ = 0.41; *p* = 0.024) and VLDL (r_s_ = 0.40; *p* = 0.030), and a negative correlation with HDL (r_s_ = − 0.56; *p* = 0.001) and LDL (r_s_ = − 0.36; *p* = 0.049). In RT patients, IL-41 showed a negative correlation with age (r_s_ = − 0.40; *p* = 0.031). In addition, raftlin showed a negative correlation with HbA1c (r_s_ = − 0.56; *p* = 0.001) and a positive correlation with HDL (r_s_ = 0.45; *p* = 0.014) (Table 3).

**Table 3:** Spearman’s rank correlation analysis of IL-29, IL-41, and raftlin with clinical characteristics in patients with type 2 diabetes mellitus.

Variable	Age	BMI	HbA1c	FPG	uACR	BUN	sCR	TC	TG	HDL	LDL	VLDL
ND	r_s_	r_s_	r_s_	r_s_	r_s_	r_s_	r_s_	r_s_	r_s_	r_s_	r_s_	r_s_
IL-29	− 0.23	0.00	0.27	0.28	0.15	− 0.06	− 0.13	0.09	0.17	− 0.34	− 0.08	0.15
IL-41	0.32	0.01	0.22	0.10	− 0.12	0.25	0.01	0.08	0.24	− 0.09	− 0.03	0.12
Raftlin	− 0.04	− 0.11	0.09	− 0.09	0.00	− 0.15	0.03	− 0.11	0.14	− 0.03	− 0.21	0.22

DM	r_s_	r_s_	r_s_	r_s_	r_s_	r_s_	r_s_	r_s_	r_s_	r_s_	r_s_	r_s_
IL-29	0.12	0.00	− 0.19	0.10	0.11	0.00	− 0.09	− 0.23	0.03	0.11	− 0.17	0.07
IL-41	− 0.27	0.20	0.26	− 0.04	− 0.02	0.31	0.01	0.07	− 0.01	0.04	− 0.17	− 0.06
Raftlin	0.06	− 0.10	− 0.16	− 0.04	− 0.19	0.29	− 0.21	− 0.24	− 0.07	0.07	− 0.30	− 0.08

PN	r_s_	r_s_	r_s_	r_s_	r_s_	r_s_	r_s_	r_s_	r_s_	r_s_	r_s_	r_s_
IL-29	− 0.28	0.02	− 0.09	− 0.05	0.20	− 0.24	− 0.29	0.11	0.26	− 0.03	− 0.38	0.32
IL-41	− 0.05	0.20	0.09	− 0.05	0.23	0.14	− 0.13	− 0.02	0.15	− 0.14	− 0.03	0.08
Raftlin	− 0.01	0.04	− 0.17	− 0.32	− 0.24	− 0.37	− 0.01	− 0.05	− 0.24	− 0.08	− 0.25	− 0.28

NE	r_s_	r_s_	r_s_	r_s_	r_s_	r_s_	r_s_	r_s_	r_s_	r_s_	r_s_	r_s_
IL-29	− 0.07	− 0.22	0.04	0.14	0.11	0.22	0.16	0.01	− 0.03	0.03	0.00	0.07
IL-41	0.14	0.41*	0.23	0.20	0.03	0.04	− 0.17	− 0.07	0.32	− 0.56**	− 0.36*	0.40*
Raftlin	− 0.12	0.11	0.12	0.18	− 0.01	0.03	− 0.19	− 0.03	− 0.14	− 0.08	− 0.06	0.02

RT	r_s_	r_s_	r_s_	r_s_	r_s_	r_s_	r_s_	r_s_	r_s_	r_s_	r_s_	r_s_
IL-29	− 0.25	− 0.09	− 0.18	0.02	0.22	− 0.06	0.14	− 0.02	0.01	0.19	0.22	0.20
IL-41	− 0.40*	0.24	− 0.20	0.15	0.27	0.03	0.16	0.05	− 0.02	0.22	0.18	− 0.02
Raftlin	− 0.15	− 0.19	− 0.56**	− 0.18	− 0.14	− 0.31	− 0.16	− 0.30	− 0.29	0.45*	− 0.01	− 0.11

ND: newly diagnosed patients (n = 34); DM: patients without diabetes-related microvascular complications (n = 30); PN: patients with peripheral neuropathy (n = 30); NE: patients with nephropathy (n = 30); RT: patients with retinopathy (n = 30); IL: interleukin; BMI: body mass index; HbA1c: glycated hemoglobin; FPG: fasting plasma glucose; UACR: urine albumin-to-creatinine ratio; BUN: Blood urea nitrogen; sCR: serum creatinine; eGFR: estimated glomerular filtration rate; TC: total cholesterol; TG: triglycerides; HDL: high-density lipoprotein; LDL: Low-density lipoprotein; VLDL: very-low-density lipoprotein. r_s_: correlation coefficient (significant correlations are indicated in bold and marked with an asterisk [*p < 0.05; **p < 0.01]).

## Discussion

Despite significant advances in the clinical management of T2DM, the underlying pathophysiological mechanisms responsible for disease-associated MICs remain incompletely understood. Circulating biomarkers have emerged as promising candidates for elucidating the pathogenesis of T2DM and predicting its associated MICs^40^; however, existing evidence remains fragmented and often inconclusive. The current study focused on recently identified serum biomarkers (IL-29, IL-41, and raftlin) and explored their association with three T2DM-related MICs (PN, NE, and RT), as studies in this context are limited.

Although IL-29 concentrations tended to be higher in T2DM patients versus HC, particularly those with ND, PN, or RT, no significant variations were observed between T2DM patients and HC or between disease groups (ND, DM, PN, NE, and RT), Moreover, ROC curve analysis and logistic analysis did not support an association between IL-29 and T2DM in patients with or without MICs. Since IL-29 is more closely associated with viral defenses and epithelial immunity than with metabolic inflammation^14^, elevated IL-29 levels are more likely to be secondary to immune activation rather than a driver of microvascular damage. However, functionally, studies have shown that IL-29 exhibits enhancing effects on the synthesis of pro-inflammatory cytokines, such as IL-6 and TNF-α, which in turn facilitate the recruitment and activation of immune cells at sites of tissue injury or infection^16^. In light of these immune capabilities and under chronic metabolic conditions, such as T2DM, persistent IL-29 signaling may contribute to sustaining low-grade inflammation and endothelial dysfunction, two key features associated with MICs in T2DM, as described in RT^41^. Recent evidence has confirmed that IL-29 concentrations were significantly higher in serum of T2DM patients with RT or neurovascular complications compared to DM patients or HC, suggesting that IL-29 may be associated with these complications^19,42^. However, conflicting results have also been reported for IL-29 in T2DM patients, especially those with foot ulcers, where serum concentrations of this cytokine were significantly lower in patients compared to HC^18^. Factors that may explain these inconsistent results include, but are not limited to, medications, obesity, and age. Mononuclear immune cells of T2DM patients, who were treated with sitagliptin (an oral hypoglycemic agent) for six months, showed lower expression levels of IL-29 versus patients not treated with sitagliptin^17^. IL-29 has also been associated with obesity-induced inflammation in mice^43^. In the current study, although no significant differences were observed, oral hypoglycemic medications were similarly associated with lower serum IL-29 concentrations in DM, PN, and RT patients compared to patients using insulin therapy, while an opposite observation was made in NE patients. Regarding obesity, O/O PN and RT patients also displayed higher IL-29 concentrations versus NW patients (Supplementary Table S1). Age may be another factor linked to IL-29 variability among T2DM patients. In RT patients, it was observed that the age range 18–50 years was associated with elevated IL-29 concentrations compared to the age range 51–68 years, and the *p*-value was close to 0.05 (16.3 vs. 6.7 pg/mL; *p* = 0.061; Supplementary Table S1). Interestingly, most RT patients were O/O and were classified as 51–68 years old (90.0 and 93.3%, respectively). These two observations suggest that obesity and aging may be predisposing factors influencing the association of IL-29 with diabetic RT, and future studies should address these considerations.

IL-41 concentrations were slightly altered across the five disease groups (ND, DM, PN, NE, and RT), and consistently high concentrations of this cytokine were observed in the serum of T2DM patients, particularly ND, DM, and NE patients, where the *pc*-value remained significant. However, ROC curve analysis demonstrated that IL-41 was statistically significant in distinguishing between T2DM patients and HC only in all patients, as well as in ND and DM groups, where the AUC value exceeding 0.70. Logistic regression analysis also indicated that higher IL-41 concentrations were associated with an OR greater than 1.0 in all patients, as well as in ND and DM groups. With respect to T2DM-related MICs (PN, NE, and RT), IL-41 did not show any statistical significance in distinguishing between patients with and without MICs, nor did it show any association with these MICs. Our results suggest that IL-41 is best described as a candidate biomarker that shows association and discriminatory ability in ND and DM groups, but not in PN, NE or RT groups. Chung and colleagues were the first to report increased concentrations of IL-41 in serum of T2DM patients. Furthermore, these concentrations were inversely correlated with cardiometabolic risk factors^44^. Wang and colleagues added, in line with the current study, that IL-41 concentrations were elevated in prediabetic and ND patients. Moreover, IL-41 concentrations showed a positive correlation with BMI, FPG, HbA1c, TG, and insulin resistance, and a negative correlation with HDL^45^. A later study reported opposite observations, with IL-41 concentrations decreased in patients with T2DM and patients with diabetic kidney disease^11^. Another study demonstrated that IL-41 levels were higher in T2DM patients with atherosclerosis than in patients without atherosclerosis^46^. These studies, although not numerous and with inconsistent findings, indicate that IL-41 is a novel adipokine believed to be associated with T2DM and related MICs and MACs. However, the mechanisms underlying the association of IL-41 with T2DM and disease-related clinical complications still need to be deciphered. Studies have indicated that M2 macrophages, which are important cells in regulating inflammation through their functional interactions with different T cell subsets, express the highest levels of IL-41. IL-41 expression in these cells has been shown to be influenced by the cytokine milieu; cytokines such as TNF-α, IL-12, and IL-17A exert stimulatory effects, while IFN-γ and TGF-β have inhibitory effects^23^. Therefore, it is hypothesized that IL-41 is involved in regulating T cell-mediated immune responses of the Th1, Th2, and Th17 types^25^. In this context, It has been shown that the proportions of Th1, Th2, and Th17 cells are altered in patients with T2DM, especially in those with NE^47^. Therefore, the association of IL-41 with T2DM can be explained through indirect effects on other immune components such as T cells and cytokines. In a mouse model of diabetic kidney disease, increased renal expression of IL-41 significantly contributed to alleviating kidney damage through downregulating effects on the TGF-β1/Smads signaling pathway^48^. Autophagy, a vital process involved in maintaining cellular homeostasis and glucose balance, is also affected by IL-41. A recent study has shown that this cytokine may ameliorate diabetic cardiomyopathy by influencing autophagy signaling pathways^49^. Accordingly, IL-41-association with T2DM and consequent clinical complications can be attributed to its diverse biological functions, particularly those related to inflammation and metabolism. The considerable heterogeneity of T2DM, in terms of multiple pathogenic pathways and diverse MICs and MACs^50^, may explain the inconsistencies in findings of studies investigating the relationship between IL-41 and T2DM.

The most interesting part of this study is raftlin, which showed a tendency for decreased serum levels in T2DM groups, with RT patients exhibiting the lowest levels, and the difference was significant before correcting the *p*-value compared to PN patients or HC. However, raftlin showed no acceptable discriminatory ability versus DM patients and was not linked to any of the MICs associated with T2DM, but interestingly, raftlin levels were positively correlated with HDL levels (r_s_ = 0.45) and negatively correlated with HbA1c levels (r_s_ = − 0.56) in RT patients. Perhaps the only available evidence linking raftlin to T2DM is that provided by Mesen and colleagues, who reported that serum raftlin levels were significantly lower in patients compared to HC. However, when examining these levels in patients with and without RT, no significant differences were observed. A negative correlation was also found between raftlin levels and glucose levels^12^. In other inflammatory conditions, such as sepsis and acute appendicitis, recent evidence has pointed to the predictive potential of raftlin in these cases, making it a promising and emerging area of ​​research in this context^28^. As a lipid raft protein involved in regulating cellular signal transduction, raftlin has been linked to the regulation of inflammatory responses through several immune pathways, particularly those involving B and T cell signaling, where raftlin has been shown to play a crucial functional role. It has been shown to play a pivotal role in regulating BCR-mediated cellular signaling, and raftlin deficiency has been associated with impaired BCR-mediated tyrosine phosphorylation, which was enhanced upon raftlin overexpression^27^. In addition, raftlin has been shown to specifically upregulate and enhance the formation of a complex with one of the key transcription factors of Th17 cells, RORγt, and disruption of this complex is associated with a numerical decrease in pathogenic Th17 cells^29^. In T2DM, significant changes in B cell function and cellular signaling pathways are evident with substantial implications for promoting inflammation due to their ability to enhance the pro-inflammatory functions of T cells, as well as their production of pro-inflammatory cytokines^51^. In addition, B cells are a key component in supporting the development of two characteristics associated with T2DM; hyperglycemia and insulin resistance^52^. Similarly, Th17 cells, which are characterized by the production of the pro-inflammatory cytokine IL-17, are dysfunctional in T2DM, thereby promoting inflammation and insulin resistance^53^. Another immunological aspect of raftlin relates to its effects on the signaling pathways of TLR3 and TLR4, which are important components of the innate immune system and play a significant role in mediating inflammatory responses by promoting the expression of IFN genes^28^. The signalling pathways of both receptors are dysregulated in T2DM and their participation in the disease pathogenesis and clinical complications has been proposed^54^. Taken together, these observations suggest that impaired raftlin production may affect several of the aforementioned immune signaling pathways, which are dysfunctional in T2DM, and thus may be indirectly linked to the disease and its complications. Studying these immune pathways in conjunction with raftlin in T2DM could contribute to a deeper understanding of the mechanisms by which raftlin is associated with this disease and/or the associated RT.

Bivariate correlation analysis revealed the degree and direction of the relationship between IL-29, IL-41, and raftlin, which varied depending on the T2DM group. Among ND patients, IL-29, IL-41, and raftlin maintained significant and consistent positive correlations, and the same was observed in RT patients, but the correlations were not significant. In PN patients, the three variables showed no significant correlations, while in NE patients, only IL-41 and raftlin showed a significant positive correlation. In DM patients, raftlin showed significant positive correlations with both IL-29 and IL-41. Despite the lack of supporting evidence, the correlation pattern between IL-29, IL-41, and raftlin was influenced by MICs associated with T2DM, and whether patients were newly diagnosed or had not experienced microvascular complications. These disease-group-dependent correlation patterns were also observed when evaluating IL-29, IL-41, and raftlin correlations with demographic, clinical, and laboratory variables. In fact, only NE and RT patients showed significant correlations: IL-41 with BMI, HDL, LDL, and VLDL in NE patients, and IL-41 with age, and raftlin with HbA1c and HDL in RT patients. The observed correlations may support the hypothesis that IL-29, IL-41, and raftlin are part of a broader inflammatory-metabolic network, warranting targeted mechanistic studies.

### Limitations

The sample size in the T2DM cohorts was relatively small, which is a major limitation, and larger longitudinal studies are needed to determine the causal relationship between IL-29, IL-41, raftlin and T2DM and related MICs. In addition, the cross-sectional case–control study design limits causal inference and does not deeply assess the true "risk of developing" MICs, and therefore additional mechanistic assessments are needed. The study groups were not age-balanced, as age may influence levels of inflammatory biomarkers. In addition, detailed information on individual antidiabetic drug classes, including SGLT2 inhibitors and GLP-1 receptor agonists, was not available for all participants. Therefore, the potential influence of these medications on IL-29, IL-41, and raftlin concentrations could not be fully evaluated.

## Conclusions

Elevated levels of IL-41 were the most consistent and statistically significant association with T2DM, particularly in ND, DM, and NE patients. Decreased levels of raftlin were observed in T2DM patients, especially those with RT, but with limited diagnostic performance and uncertain biological significance. IL-29 levels did not demonstrate robust group differences among T2DM patients. The observed alterations in these biomarkers likely reflect immune-metabolic dysregulation; however, their functional role in T2DM and associated MICs remains unclear. Therefore, IL-29, IL-41, and raftlin role in inflammation, metabolic dysfunction, and T2DM-related MICs requires further investigation through prospective longitudinal and mechanistic studies.

## Supplementary Information

Below is the link to the electronic supplementary material.


Supplementary Material 1


## Data Availability

The datasets generated and/or analysed during the current study are not publicly available due to the confidentiality of patient attributes, but are available from the corresponding author upon reasonable request, with anonymised datasets.
