# Supplementary material for: Serum profile of IL-29, IL-41, and raftlin in microvascular complications of type 2 diabetes mellitus
Source: Sci Rep. 2026 Jun 16;16:27465. doi: 10.1038/s41598-026-55654-y (PMC13534468; doi:10.1038/s41598-026-55654-y)
Supplement: Supplementary file 1 [file 41598_2026_55654_MOESM1_ESM.pdf]

Supplementary Table S1: IL-29, IL-41, and raftlin levels classified according to T2DM characteristics.

ND		IL-29; pg/mL			IL-41; ng/mL			Raftlin; pg/mL		
		Median	IQR: 25%	IQR: 75%	Median	IQR: 25%	IQR: 75%	Median	IQR: 25%	IQR: 75%
Age; year	18-50	8.2	5.2	12.1	7.5	6.8	8.0	39.8	33.5	56.7
	51-68	7.1	5.5	9.6	8.7	7.3	9.0	42.1	38.6	59.4
	p -value	0.362			0.138			0.484		
Gender	Male	8.3	3.7	10.6	7.3	6.8	8.8	40.4	29.2	58.0
	Female	7.5	6.6	9.9	7.8	7.2	8.8	41.8	38.0	58.0
	p -value	0.986			0.535			0.317		
BMI	NW	7.2	6.6	7.9	6.8	6.4	7.7	40.9	38.0	47.6
	O/O	7.7	5.2	11.2	7.6	7.1	8.8	41.5	34.6	59.4
	p -value	0.395			0.166			0.752		
Family history	Yes	8.3	5.9	10.6	7.4	6.8	9.0	41.5	34.6	58.0
	No	7.1	4.3	9.9	7.7	7.3	8.3	40.7	30.2	58.0
	p -value	0.278			0.726			0.739		
Medication	Oral	NA	NA	NA	NA	NA	NA	NA	NA	NA
	Insuline	NA	NA	NA	NA	NA	NA	NA	NA	NA
	p -value	NA	NA	NA	NA	NA	NA	NA	NA	NA
**DM**		**IL-29; pg/mL**			**IL-41; ng/mL**			**Raftlin; pg/mL**		
		**Median**	**IQR: 25%**	**IQR: 75%**	**Median**	**IQR: 25%**	**IQR: 75%**	**Median**	**IQR: 25%**	**IQR: 75%**
Age; year	18-50	6.1	3.5	10.0	7.4	6.9	8.4	41.5	30.8	56.0
	51-68	9.3	4.6	10.9	7.2	6.4	7.6	39.8	33.5	61.4
	p -value	0.983			0.471			0.641		
Gender	Male	5.9	4.1	9.8	7.4	6.9	9.1	43.0	31.9	56.1
	Female	8.3	5.0	14.2	7.3	6.9	7.7	40.4	33.5	58.7
	p -value	0.519			0.647			0.901		
BMI	NW	6.2	1.5	10.9	6.8	5.9	7.6	55.1	50.8	59.4
	O/O	6.7	4.8	10.5	7.3	6.9	8.3	40.6	32.1	58.0
	p -value	0.739			0.506			0.280		
Family history	Yes	7.5	5.2	10.0	7.2	6.9	8.5	40.9	34.0	59.4
	No	6.5	3.2	12.5	7.4	7.0	7.6	43.4	31.3	57.4
	p -value	0.767			1.000			0.816		
Medication	Oral	3.5	1.5	9.4	7.6	6.9	8.4	40.4	29.2	50.8
	Insuline	9.0	5.4	10.9	7.3	6.9	8.2	41.5	34.0	60.0
	p -value	0.082			0.844			0.418		
**PN**		**IL-29; pg/mL**			**IL-41; ng/mL**			**Raftlin; pg/mL**		
		**Median**	**IQR: 25%**	**IQR: 75%**	**Median**	**IQR: 25%**	**IQR: 75%**	**Median**	**IQR: 25%**	**IQR: 75%**
Age; year	18-50	9.4	8.3	11.6	6.8	5.9	7.6	61.1	46.7	68.4
	51-68	5.5	3.7	11.6	7.0	5.8	7.6	46.7	38.0	61.4
	p -value	0.170			0.982			0.202		
Gender	Male	8.5	4.8	11.6	7.1	5.8	7.7	47.0	38.0	61.4
	Female	6.4	3.7	15.0	6.7	5.2	7.5	50.8	43.3	69.1
	p -value	0.914			0.491			0.561		
BMI	NW	6.0	4.1	9.3	5.6	5.2	7.3	46.7	38.0	48.3
	O/O	8.7	4.4	12.6	7.0	6.5	7.6	50.5	42.4	69.5
	p -value	0.351			0.146			0.406		
Family history	Yes	6.4	3.5	10.2	6.9	6.2	7.6	46.4	38.0	50.2
	No	9.6	5.5	13.1	6.7	5.8	7.5	61.4	48.3	69.9
	p -value	0.155			0.933			**0.040**		
Medication	Oral	6.6	3.9	10.7	7.0	6.3	7.5	49.2	40.7	68.5
	Insuline	9.3	4.8	15.0	6.9	5.2	7.9	47.0	41.5	66.2
	p -value	0.220			0.852			0.771		
**NE**		**IL-29; pg/mL**			**IL-41; ng/mL**			**Raftlin; pg/mL**		
		**Median**	**IQR: 25%**	**IQR: 75%**	**Median**	**IQR: 25%**	**IQR: 75%**	**Median**	**IQR: 25%**	**IQR: 75%**

**Age; year**	**18-50**	7.7	2.2	10.0	7.5	6.5	10.7	44.6	39.2	65.5
	**51-68**	6.6	2.9	7.7	7.7	7.0	10.2	39.2	34.6	56.0
	p **-value**	0.355			0.983			0.504		
**Gender**	**Male**	7.5	4.4	9.9	7.6	6.8	10.3	45.2	38.9	65.2
	**Female**	4.6	2.0	7.4	7.5	7.0	12.9	38.6	32.4	40.9
	p **-value**	0.288			0.917			0.253		
**BMI**	**NW**	8.2	6.6	10.0	7.1	5.9	7.8	34.6	10.6	52.7
	**O/O**	7.1	2.2	9.9	7.7	7.0	10.4	40.9	38.6	65.5
	p **-value**	0.426			0.284			0.226		
**Family history**	**Yes**	7.2	2.2	9.9	7.7	6.5	10.7	40.9	39.2	65.5
	**No**	7.1	2.9	8.2	7.3	7.0	10.2	39.8	32.4	56.0
	p **-value**	0.780			0.949			0.561		
**Medication**	**Oral**	7.1	4.4	9.9	7.7	6.8	10.4	39.8	36.0	65.2
	**Insuline**	4.8	1.2	8.2	7.4	7.1	8.0	44.6	39.2	52.7
	p **-value**	0.568			0.717			0.640		

**RT**		**IL-29; pg/mL**			**IL-41; ng/mL**			**Raftlin; pg/mL**		
		**Median**	**IQR: 25%**	**IQR: 75%**	**Median**	**IQR: 25%**	**IQR: 75%**	**Median**	**IQR: 25%**	**IQR: 75%**
**Age; year**	**18-50**	16.3	11.8	20.9	8.2	7.3	9.0	60.7	59.4	62.1
	**51-68**	6.7	4.8	10.5	6.7	6.1	7.3	37.4	27.9	52.4
	p **-value**	0.061			0.096			0.124		
**Gender**	**Male**	9.3	5.0	10.9	6.5	6.0	7.2	37.4	30.2	56.0
	**Female**	5.9	4.3	10.9	7.2	6.6	8.2	41.2	27.4	56.4
	p **-value**	0.641			0.117			0.899		
**BMI**	**NW**	3.5	1.5	10.9	6.5	5.1	8.0	40.9	31.9	48.9
	**O/O**	9.0	5.2	11.6	6.9	6.2	7.3	37.4	26.6	59.4
	p **-value**	0.254			0.809			0.917		
**Family history**	**Yes**	9.2	5.0	11.7	6.7	6.1	7.3	41.8	31.0	57.7
	**No**	5.6	5.0	9.3	7.2	6.2	8.3	31.9	25.6	41.5
	p **-value**	0.276			0.468			0.312		
**Medication**	**Oral**	7.4	5.5	10.0	7.2	6.0	8.0	37.4	29.2	56.0
	**Insuline**	9.0	4.6	11.8	6.5	6.2	7.3	42.7	25.6	59.4
	p **-value**	0.901			0.351			0.771		

T2DM: Type 2 diabetes mellitus; ND: Newly diagnosed patients (n = 34); DM: Patients without diabetes-related microvascular complications (n = 30); PN: Patients with peripheral neuropathy (n = 30); NE: Patients with nephropathy (n =30); RT: Patients with retinopathy (n = 30); IL: Interleukin; BMI: Body mass index; NW: Normal weight; O/O:Overweight/obese; NA: Not applicable; *p*: Two-tailed probability (significant *p*-value is indicated in bold). Significance was determined using Mann-Whitney U test.
